# Echocardiographic and invasive RV-PA coupling predict mortality after edge-to-edge repair for degenerative mitral regurgitation, while pulmonary artery pulsatility index does not: a single-center observational study

**DOI:** 10.1007/s00392-025-02795-1

**Published:** 2025-12-09

**Authors:** Lara Waldschmidt, Christoph Pauschinger, Benedikt Koell, Jana Doering, Johannes Schirmer, Simon Pecha, Sebastian Ludwig, Jessica Weimann, Leona Steinhoff, Lydia Plewe, Andreas Schaefer, Hermann Reichenspurner, Stefan Blankenberg, Niklas Schofer, Daniel Kalbacher

**Affiliations:** 1 Department of General an Interventional Cardiology, University Heart and Vascular Center Hamburg, University Medical Center Hamburg-Eppendorf , Hamburg, Germany; 2 German Center for Cardiovascular Research, Partner Site, Hamburg/Luebeck/Kiel, Germany; 3 Department of Cardiovascular Surgery, University Heart and Vascular Center Hamburg, University Medical Center Hamburg-Eppendorf, Hamburg, Germany

**Keywords:** Degenerative mitral regurgitation, RV-PA coupling, Pulmonary artery pulsatility index, Edge To Edge repair

## Abstract

**Background:**

Right ventricular–pulmonary artery coupling and the pulmonary artery pulsatility index (PAPi) have emerged as potential prognostic markers in cardiovascular disease. Their utility in patients with degenerative mitral regurgitation (DMR) undergoing transcatheter mitral edge-to-edge repair (M-TEER) remains unclear.

**Objectives:**

To evaluate the prognostic value of RV-PA coupling and PAPi in DMR patients treated with M-TEER.

**Methods:**

This retrospective single-center study included 293 patients with DMR undergoing M-TEER between 2012 and 2022. RV-PA coupling was assessed by echocardiographic tricuspid annular plane systolic excursion (TAPSE) to systolic pulmonary artery pressure (sPAP_echo_) and, in a subset, invasively (TAPSE/sPAP_invasive_). PAPi was calculated as (sPAP—diastolic PAP) / right atrial pressure. The primary endpoint was all-cause mortality at 2 years; the secondary endpoint was a composite of mortality or heart failure hospitalization.

**Results:**

RV-PA uncoupling (TAPSE/sPAP_echo_ < 0.307 mm/mmHg) was associated with higher 2-year mortality (52.2% vs. 22.2%; HR = 2.64; *p* < 0.001) and composite events (56.4% vs. 30.1%; HR = 2.22; *p* = 0.0023). Similar results were observed with invasively derived TAPSE/sPAP (cut-off < 0.315 mm/mmHg). In contrast, PAPi < 3.65 was not associated with outcomes. TAPSE alone showed no prognostic significance.

**Conclusion:**

RV-PA coupling, but not PAPi, predicts mortality and heart failure rehospitalization following M-TEER in DMR patients. These findings highlight the value of RV-PA coupling indices for risk stratification and support their routine use in preprocedural assessment.

**Graphical Abstract:**

**Figure:**
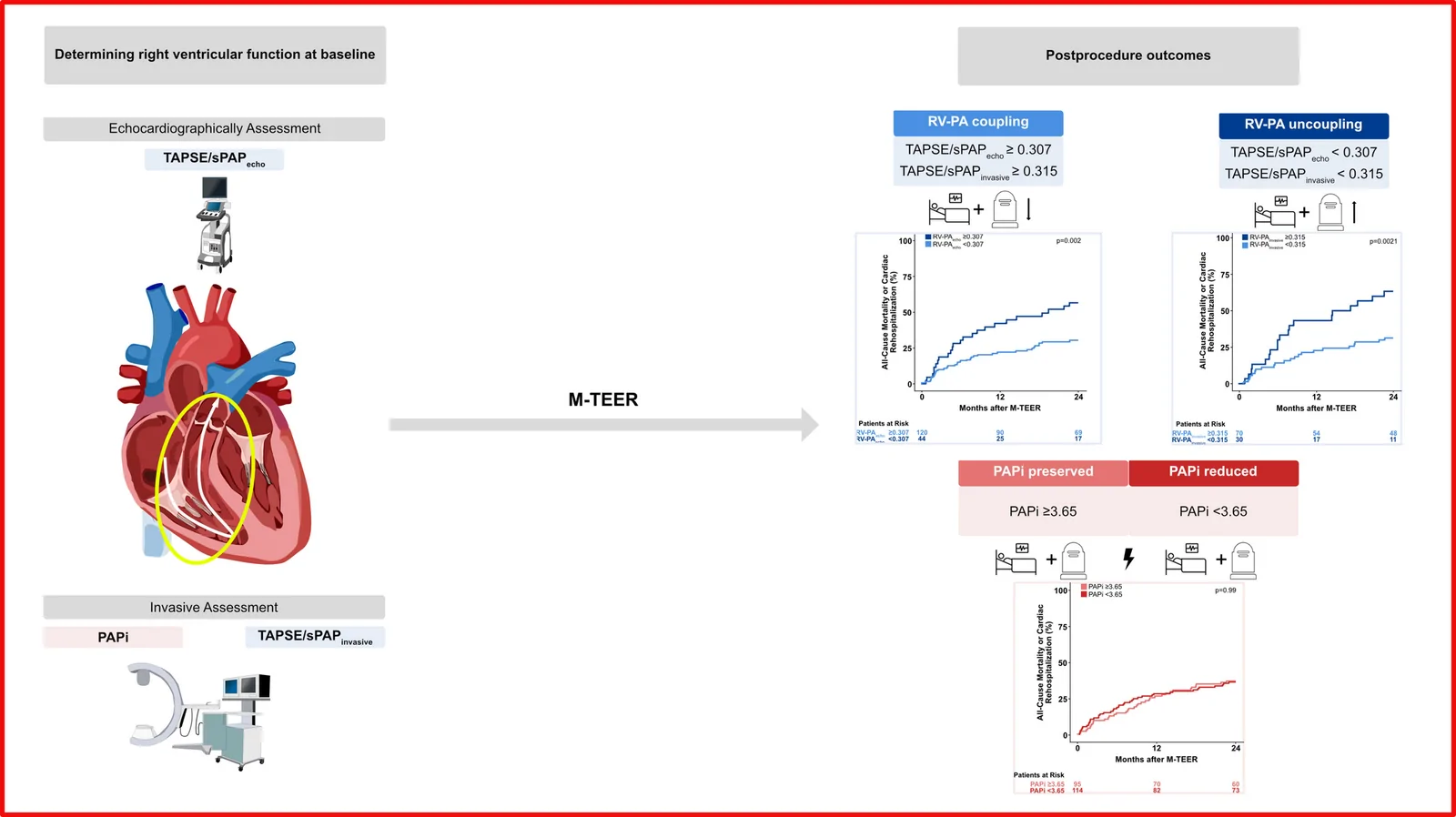

**Supplementary Information:**

The online version contains supplementary material available at https://doi.org/10.1007/s00392-025-02795-1.

## Introduction

Degenerative mitral regurgitation (DMR) remains one of the most prevalent valvular heart diseases, particularly affecting elderly populations [1]. Transcatheter mitral edge-to-edge repair (M-TEER) has become an important treatment option for high surgical risk patients, offering an effective and less invasive alternative to open-heart surgery [2]. However, risk stratification remains challenging, and conventional clinical and echocardiographic parameters have limited predictive accuracy for postprocedural outcomes.

Right ventricular (RV) dysfunction is increasingly recognized as a key prognostic factor across a range of cardiovascular conditions including heart failure [3], pulmonary hypertension [4], mitral regurgitation (MR) [5, 6], and tricuspid regurgitation (TR) [7]. In DMR, chronic volume and pressure overload may induce pulmonary hypertension and ultimately impair RV function [8]. While isolated echocardiographic markers such as tricuspid annular plane systolic excursion (TAPSE) are widely used, they often fail to capture the dynamic interplay between RV contractility and afterload [9]. In this context, the concept of RV-pulmonary artery (PA) coupling has gained attention as an integrative metric of RV performance, combining contractile reserve and afterload adaptation [10]. Noninvasively, RV-PA coupling can be estimated by the TAPSE-to-systolic pulmonary artery pressure (sPAP) ratio, which has demonstrated prognostic value in pulmonary hypertension [10], heart failure with preserved ejection fraction [11], tricuspid regurgitation [12, 13], aortic stenosis [14] and functional mitral regurgitation [15, 16].

However, its role in DMR, particularly in patients undergoing M-TEER, is less well defined. The pulmonary artery pulsatility index (PAPi), derived invasively as the difference between systolic and diastolic PAP divided by right atrial pressure, has been proposed as an additional marker of RV function (17, 18). Initially validated in acute right heart failure and advanced heart failure populations, its utility in chronic left-sided valvular heart disease remains uncertain.

This study aims to determine the prognostic value of RV-PA coupling and PAPi in patients undergoing M-TEER for DMR. By comparing both echocardiographic and invasive assessments of RV-PA coupling and evaluating PAPi in parallel, identification of best-performing predictive parameter for adverse postinterventional outcomes is seeked. The herein examined hypothesis therefore implies that impaired RV-PA coupling—rather than PAPi—is a more robust predictor of mortality and heart failure hospitalization in this patient population.

## Methods

### Study design and population

This retrospective single-center study included 293 patients DMR who underwent M-TEER between 2012 and 2022 at a tertiary care center. All patients were evaluated by an interdisciplinary heart team to determine treatment eligibility in accordance with current European guidelines [2]. M-TEER was performed using either the MitraClip System (Abbott, Santa Clara, California, US) or the PASCAL device (Edwards Lifesciences, Irvine, California, US).

Clinical, procedural, and follow-up data were collected from electronic medical records and entered into a dedicated database. Follow-up data were obtained via structured outpatient visits, correspondence from referring physicians, and hospital discharge summaries. The study protocol conformed to the Declaration of Helsinki, and written informed consent was obtained from all participants.

### Echocardiographic assessment

Baseline and postprocedural transthoracic echocardiography were performed by experienced physicians. MR and TR were graded using a 4-point scheme. For MR, this included a category of moderate-to-severe MR (grade 3 +, EROA 0.30–0.39 cm^2^), in line with the echocardiographic society recommendation [17], and consistent with previously published M-TEER cohorts (e.g., Doldi et al. [18]). For consistency, the same 4-point grading scheme was also applied to TR severity. TAPSE and sPAP were measured using standard views. sPAP was estimated by peak TR velocity and adding an estimated right atrial pressure (RAP) based on inferior vena cava diameter and respiratory variability [19].

RV-PA coupling was defined as the ratio of TAPSE to sPAP (TAPSE/sPAP), referred to as RV-PA_echo_. A TAPSE/sPAP ratio < 0.307 mm/mmHg was considered indicative of RV-PA uncoupling, based on published thresholds in M-TEER populations [18].

### Hemodynamic assessment

Right heart catheterization (RHC) was performed in a subset of patients before and after the procedure, following local protocols. Systolic, diastolic, mean PAP, RAP, and pulmonary capillary wedge pressure (PCWP) were measured. sPAP was used to derive the invasive TAPSE/sPAP ratio (RV-PA_invasive_), and the optimal cut-off for RV-PA uncoupling was identified via Cox regression analysis.

PAPi was calculated as (sPAP – dPAP) divided by RAP. A cut-off of < 3.65 was used to define reduced PAPi, based on prior data in heart failure populations [20].

### Endpoints

The primary endpoint was all-cause mortality at 2 years. The secondary endpoint was a composite of all-cause mortality or rehospitalization for heart failure (HFH) during the follow-up. Outcomes were stratified according to RV-function, defined by RV-PA_echo_, RV-PA_invasive_, and PAPi values.

### Statistical analysis

Continuous variables are presented as medians with interquartile ranges (IQR), and categorical variables as absolute numbers and percentages. Group comparisons were performed using the Wilcoxon rank-sum test for continuous variables and the Chi-squared test for categorical variables.

Median follow-up time is calculated with reverse Kaplan–Meier estimator. Survival analyses were conducted using Kaplan–Meier estimates, with between-group differences evaluated using log-rank test. The optimal cutoff values for RV-PA_invasive_ coupling were derived based on Cox proportional hazard models [21]. Cox regression analysis is conducted and corresponding Hazard Ratio (HR) and 95% confidence intervals (CI) are given. A two-sided *p*-value < 0.05 was considered statistically significant. Statistical significance was defined as a two-sided *p*-value of less than 0.05. All analyses were conducted using R version 4.4.3.

## Results

### Study population

**Fig. 1:**
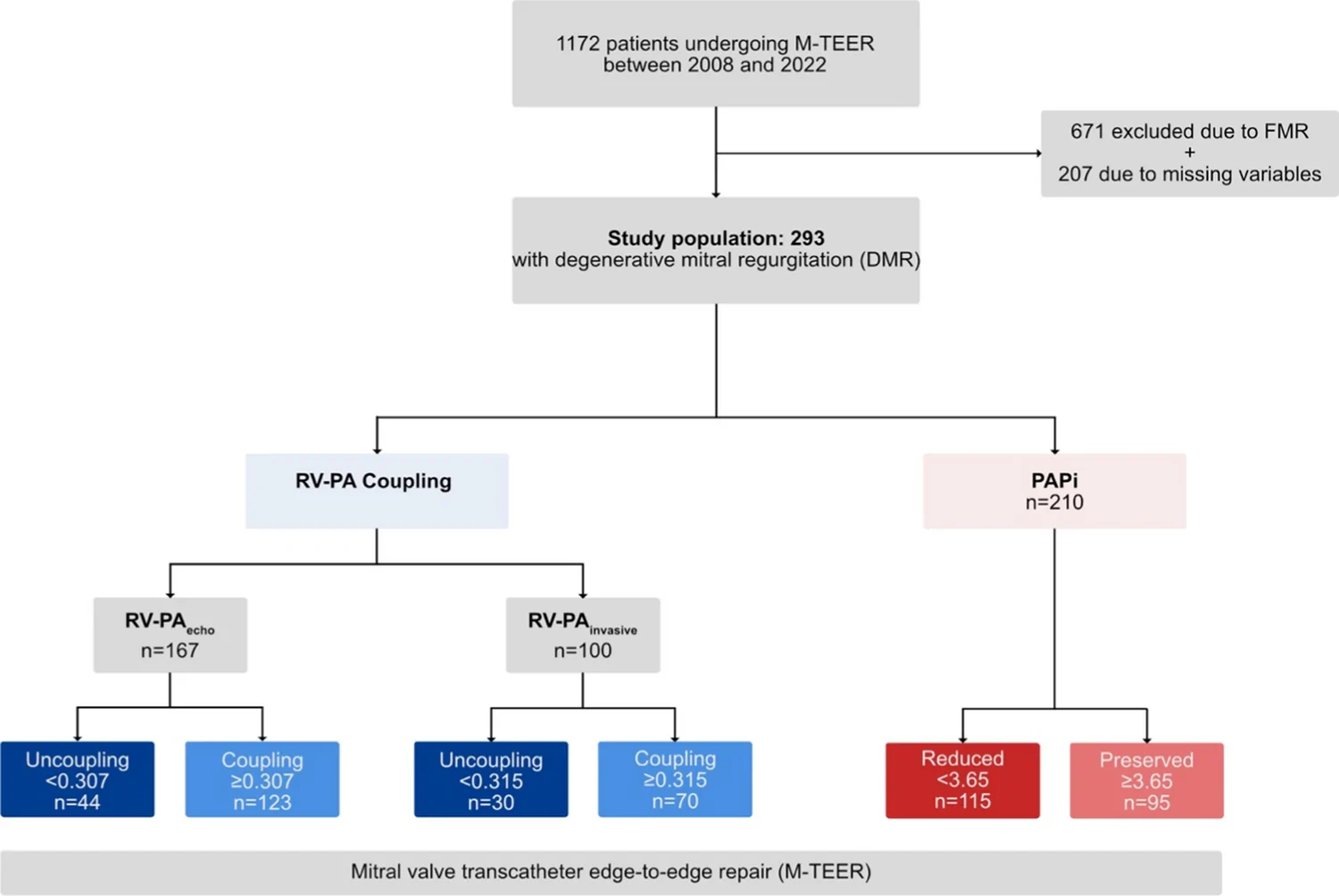
Study flow diagram: A total of 293 patients constitutes the study population of DMR patients undergoing M-TEER. RV-function was either stratified by RV-PA coupling assessed by TTE in 167 patients or by RHC in 100 patients, respectively. Uncoupling was defined at a threshold of < 0.307 and < 0.315, respectively. 210 patients had available data to determine RV-function via PAPi using an established cut-off value of < 3.65. DMR = degenerative mitral regurgitation; FMR = functional mitral regurgitation; M-TEER = mitral valve transcatheter edge-to edge repair; PAPi = pulmonary artery pulsatility index; RV-PA coupling = right ventricular pulmonary artery coupling

A total of 293 patients with DMR underwent M-TEER between 2012 and 2022 and were included in the final analysis (Fig. 1). The median age was 81.0 years (interquartile range [IQR]: 76.9–84.2), and 52.2% were male. Most patients were symptomatic, with 87.5% classified as New York Heart Association (NYHA) functional class III or IV. Comorbidities included arterial hypertension (84.2%), atrial fibrillation (79.1%), coronary artery disease (65.9%), diabetes mellitus (16.7%), and chronic kidney disease (median glomerular filtration rate [eGFR] 47.5 ml/min/1.73m^3^). The median STS-Score was 3.9% (IQR: 2.7–5.4), reflecting an increased surgical risk-cohort (Table S1).

### Baseline characteristics according to RV-PA_echo_

Echocardiographic assessment of RV-PA_echo_ coupling was available in 167 patients. Among them, 123 patients (73.7%) had preserved RV-PA coupling, and 44 patients (26.3%) showed uncoupling. Patients with RV-PA_echo_ uncoupling had a higher symptom burden, with 97.6% in NYHA class III or IV compared to 81.7% in the preserved coupling group (*p* = 0.025). They also had a higher prevalence of prior coronary artery bypass grafting (33.3% vs. 14.4%, *p* = 0.031) and numerically elevated NT-proBNP levels (4,032 vs. 2,728 pg/ml; *p* = 0.059). There was no difference in baseline MR severity between RV-PA_echo_ groups (severe MR: 81.5% vs. 79.6%; *p* = 0.95, Table 1). There was a trend toward more frequent severe TR in the uncoupled group (21.4% vs. 8.9%; *p* = 0.065).

**Table 1:** Study baseline characteristics according to RV-PA_echo_ status

	All patients (n = 167)	RV-PA Echo ≥ 0.307 (n = 123)		RV-PA Echo < 0.307 (n = 44)	*p*-value
**Clinical data**					
Male sex, n (%)	82 (49.1)		61 (49.6)	21 (47.7)	0.97
Age, years	81.0 [76.9–84.7]		81.0 [76.9–84.4]	81.7 [76.8–85.4]	0.58
BMI, kg/cm^2^	25.0 [22.8–27.9]		25.0 [22.7–27.9]	24.7 [23.1–27.9]	0.93
STS-Score, %	3.9 [2.8–5.7]		3.9 [3.0–5.6]	4.1 [2.5–6.4]	0.91
NYHA functional class					
I	2 (1.3)		2 (1.8)	-	0.94
II	19 (12.7)		18 (16.5)	1 (2.4)	0.042
III	98 (65.3)		68 (62.4)	30 (73.2)	0.30
IV	31 (20.7)		21 (19.2)	10 (24.4)	0.64
**Cardiovascular risk factors**					
AHT, n (%)	127 (87.0)		95 (89.6)	32 (80.0)	0.21
Dyslipidemia, n (%)	60 (48.0)		47 (52.2)	13 (37.1)	0.91
Nicotine abuse**,** n (%)	29 (28.0)		20 (27.0)	9 (30.0)	0.95
DM, n (%)	32 (20.7)		24 (21.4)	8 (18.6)	0.87
**Comorbidities**					
CAD, n (%)	103 (67.3)		76 (67.9)	27 (65.9)	0.97
ICM, n (%)	40 (39.6)		30 (42.3)	10 (33.3)	0.54
Previous MI, n (%)	15 (12.5)		11 (12.8)	4 (11.8)	1.00
Previous PCI, n (%)	63 (46.3)		46 (47.4)	17 (43.6)	0.83
Previous CABG, n (%)	25 (19.8)		13 (14.4)	12 (33.3)	0.031
Previous ICD/CRT	20 (16.7)		14 (15.9)	8 (23.5)	0.32
COPD, n (%)	32 (20.3)		24 (20.9)	8 (18.6)	0.93
Previous Stroke, n (%)	16 (12.9)		14 (15.9)	2 (5.56)	0.21
Afib	125 (79.1)		89 (77.4)	36 (83.7)	0.51
GFR (ml/min for 1.73 m^2^)	47.5 [32.4–62.8]		47.1 [30.8–62.6]	48.7 [37.1–62.8]	0.55
NT-proBNP (pg/ml)	3112 [1529–4912]		2728 [1529–4912]	4032 [2239–5271]	0.059
**Echocardiographic data**					
LVEF, %	54.6 [45.9–60.1]		55.0 [48.9–60.1]	53.6 [40.5–58.9]	0.23
LVESV (Simpsons), ml	48.8 [35.0–70.1]		48.0 [34.7–70.3]	49.6 [34.7–70.2]	0.98
LVEDV (Simpsons), ml	106.3 [81.4–141.5]		107.0 [89.9–140.7]	101.8 [77.1–146.6]	0.41
TAPSE, mm	18.0 [15.0–22.0]		19.0 [17.0–23.0]	14.0 [11.0–16.0]	< 0.001
sPAP_**echo**_, mmHg	42.0 [34.0–55.0]		38.5 [32.0–49.8]	59.5 [49.4–68.1]	< 0.001
MR grade					
2 +	-		-	-	-
3 +	31 (19.0)		22 (18.5)	9 (20.5)	0.95
4 +	132 (81.0)		97 (81.5)	35 (79.6)	0.95
TR grade					
0 +	-		-	-	-
1 +	53 (34.1)		44 (38.9)	9 (21.4)	0.064
2 +	52 (33.6)		36 (31.9)	16 (38.1)	0.59
3 +	31 (20.0)		23 (20.4)	8 (19.1)	1.00
4 +	19 (12.3)		10 (8.6)	9 (21.4)	0.065
**Invasive Data**					
sPAP_RHC_, mmHg	47.0 [39.0–54.0]		44.5 [38.0–53.0]	48.5 [41.8–61.2]	0.054
dPAP_RHC_, mmHg	19.0 [14.0–22.0]		17.5 [14.0–20.6]	20.0 [Des.4–23.0]	0.082
mPAP_RHC_, mmHg	29.0 [24.4–34.6]		28.0 [23.0–33.0]	31.0 [26.4–36.7]	0.054
mPCWP_RHC_, mmHg	18.0 [14.0–23.0]		16.0 [12.4–22.0]	21.0 [16.8–26.0]	0.001
mLAP_RHC_, mmHg	16.0 [12.0–22.0]		14.0 [11.7–20.0]	19.0 [15.7–24.3]	0.002
CVP_RHC_, mmHg	8.0 [6.0–11.0]		8.0 [6.0–11.0]	8.5 [6.4–13.6]	0.37

*AHT* = arterial hypertension; *Afib* = atrial fibrillation, *BMI* = body mass index; *CABG *= coronary artery bypass graft, *CAD* = coronary artery disease, *COPD* = chronic obstructive pulmonary disease, *CRT* = cardiac resynchronization therapy, *CVP* = central venous pressure, *DM* = diabetes mellitus, *dPAP* = diastolic pulmonary artery pressure, echo = echocardiography, *GFR* = glomerular filtration rate, *ICD* = implantable cardioverter-defibrillator, *ICM* = ischemic heart disease, *LVEDV* = left ventricular diastolic volume, *LVEF* = left ventricular ejection fraction, *LVESV* = left ventricular systolic volume, *MI* = myocardial infarction, *mLAP* = mean left atrial pressure, mPAP = mean pulmonary artery pressure, *mPCWP* = pulmonary capillary wedge pressure, *MR* = mitral regurgitation, *NT-proBNP* = N-terminal pro-b-type natriuretic peptide, *NYHA* = New York Heart Association, *PCI* = percutaneous coronary intervention, *RHC* = right heart catheter, *sPAP* = systolic pulmonary artery pressure, *STS Score* = Society of Throacic Surgeons Score, *TAPSE *= tricuspid annular plane systolic excursion, *TR* = tricuspid regurgitation,

Hemodynamically, these patients also showed significantly higher left-sided filling pressures. The median mean left atrial pressure (LAP) was 19.0 mmHg in the RV-PA_echo_ uncoupled compared to 14.0 mmHg in the RV-PA_echo_ coupled group (*p* = 0.002), and the mean PCWP was 21.0 mmHg vs. 16.0 mmHg, respectively (*p* = 0.01; Table 1).

### Procedural outcomes according to RV-PA_echo_

**Fig. 2:**
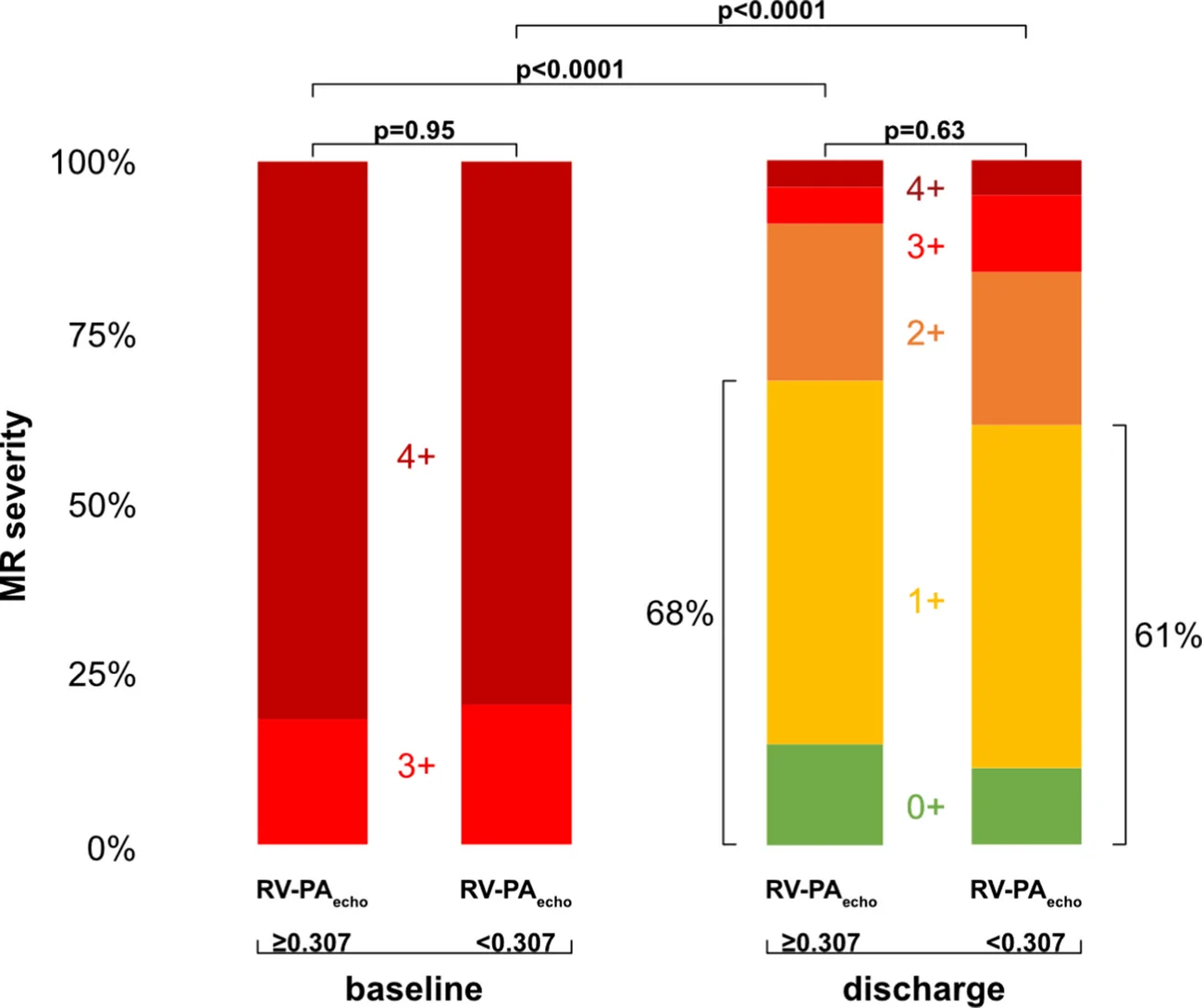
MR severity at baseline and discharge stratified by RV-PA coupling via echo: While baseline MR severity did not differ in patients with RV-PA_echo_ ≥ 0.307 and RV-PA_echo_ < 0.307, MR severity at discharge was more pronounced in patients with PV-PA_echo_ < 0.307 (< 2 + in 70% in patients with RV-PA_echo_ ≥ 0.307 vs. 64.% in patients PV-PA_echo_ < 0.307, *p* = 0.63). MR = mitral regurgitation; RV-PA_echo_ = right ventricular pulmonary artery coupling assessed by TTE, TTE = transthoracic echocardiography

MR severity was reduced to ≤ 2 + in 88.6% of patients. Procedural success, defined as MR reduction to < 2 +, was numerically lower in the RV-PA_echo_ uncoupled group (61.4% vs. 67.8%; *p* = 0.63; Fig. 2). Postprocedural trans-mitral mean gradients were significantly higher in the RV-PA_echo_ uncoupled group (4.0 mmHg [IQR: 3.0, 5.0 mmHg]) than in the RV-PA_echo_ coupled group (3.0 mmHg [IQR: 3.0, 5.0 mmHg], *p* = 0.02). Procedural complications were comparable between groups.

### Prognostic impact of RV-PA_echo_

**Fig. 3:**
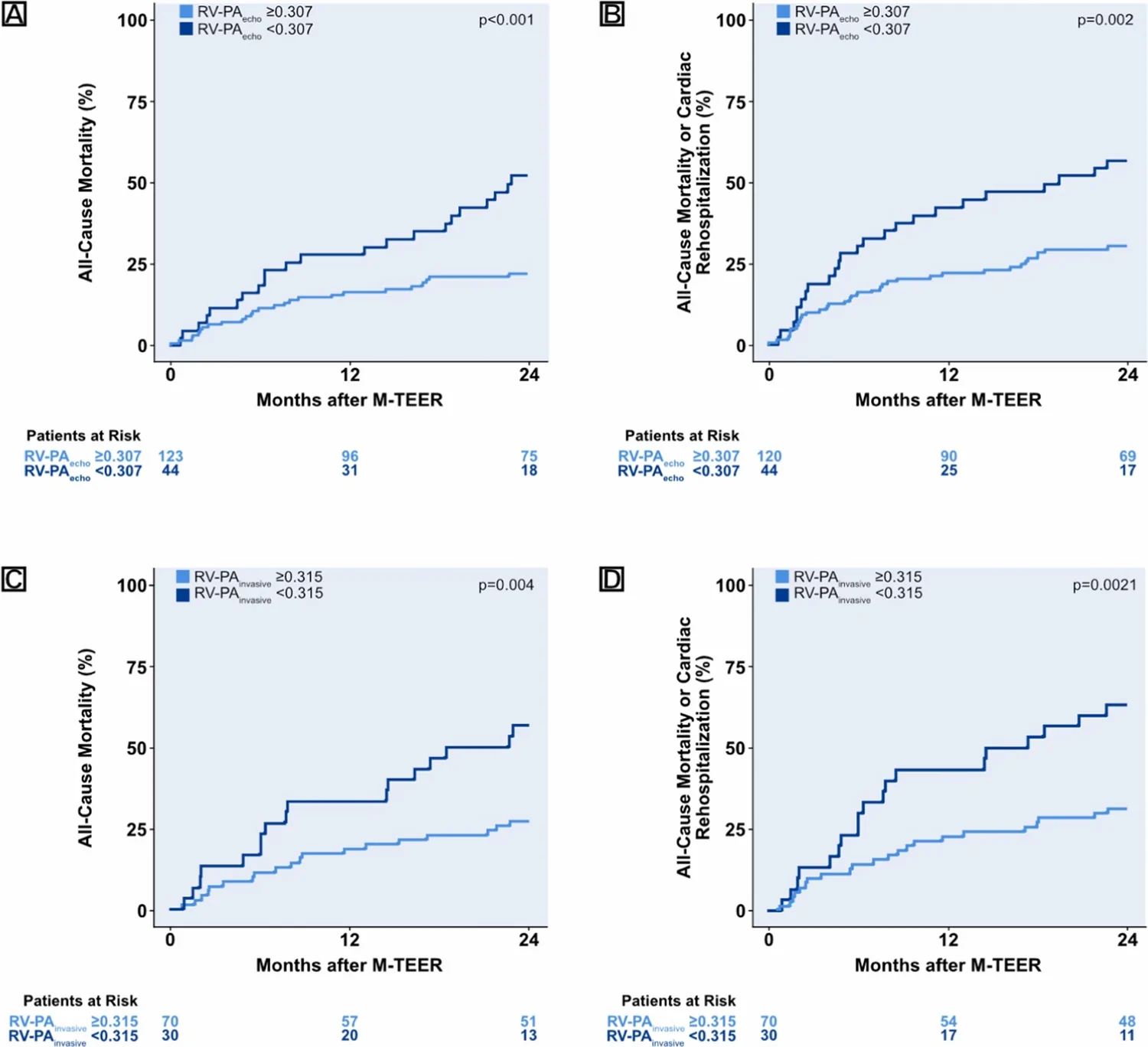
Impact of RV-PA uncoupling (TTE and RHC) on 2-year mortality and HFH: Patients with RV-PA uncoupling assessed by TTE (RV-PA_echo_ < 0.307) showed significantly reduced 2-year survival rates (**A**) and, likewise, higher rates of the composite endpoint of all-cause mortality and HFH (**B**) after M-TEER. Patients with RV-PA uncoupling assessed by RHC (RV-PA_invasive_ < 0.315) also demonstrated significantly reduced 2-year survival **(C**) and increased rates of the composite endpoint (**D**) following M-TEER. HFH = heart failure hospitalization; M-TEER = mitral valve transcatheter edge-to-edge repair; RV-PA_echo_ = right ventricular-pulmonary artery coupling assessed by echocardiography; TTE = transthoracic echocardiography

During a median follow-up of 4.52 years, the 2-year all-cause mortality rate was 28.5%. Patients with RV-PA_echo_ uncoupling had a higher 2-year mortality than those with preserved coupling (52.2% vs. 22.2%; hazard ratio [HR] = 2.64, 95%-CI = 1.50; 4.66; *p* < 0.001). They also had in increased incidence of the composite endpoint of mortality or HFH (56.4% vs. 30.1%; HR = 2.22, 95%-CI = 1.32; 3.75 *p* = 0.002; Fig. 3A and B).

### Prognostic impact of RV-PA_invasive_

In 100 patients, invasive RV-PA coupling (TAPSE/sPAP_invasive_) was assessed via right heart catheterization (baseline characteristics are summarized in Supplementary Table 2). The mean sPAP_invasive_ was 5.0 mmHg higher than the echocardiographic estimate (sPAP_echo_). Cox regression identified a TAPSE/sPAP_invasive_ threshold of < 0.315 mm/mmHg as optimal cut-off value for prognostic discrimination. Patients below this threshold showed higher 2-year mortality (56.7% vs. 27.1%; HR = 2.54, 95%-CI = 1.31; 4.90; *p* = 0.0056; Fig. 3C) and higher rates of the composite endpoint (63.3% vs. 31.4%; HR = 2.20, 95%-CI: 1.18; 4.09, *p* = 0.013; Fig. 3D).

### Prognostic value of PAPi and TAPSE alone

**Fig. 4:**
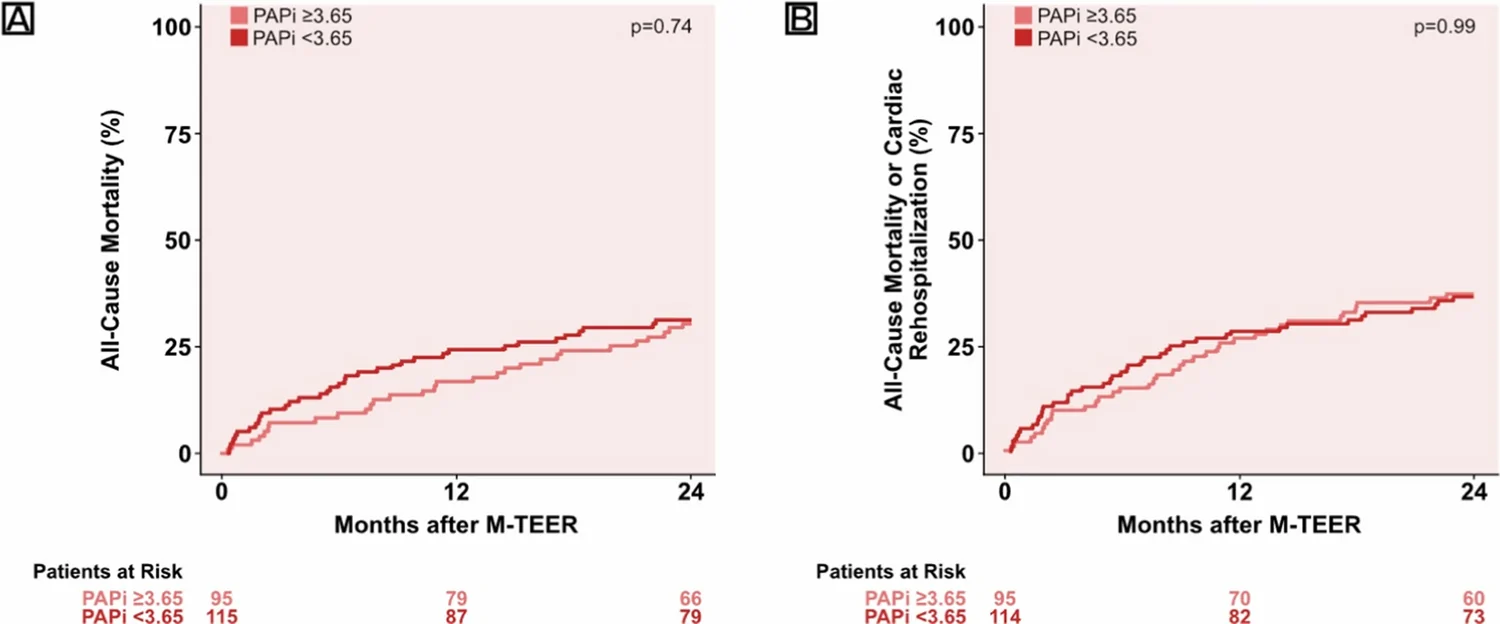
Impact of PAPi on 2-year mortality and HFH: Patients with reduced PAPi (PAPi < 3.65) did not show significantly lower 2-year survival (**A**) or a higher risk of the composite endpoint of all-cause mortality and HFH (**B**) after M-TEER. HFH = heart failure hospitalization; M-TEER = mitral valve transcatheter edge-to-edge repair; PAPi = pulmonary artery pulsatility index

Among 210 patients with available PAPi data (baseline characteristics are summarized in Supplementary Table 3), PAPi < 3.65 was not associated with increased mortality (30.5% vs. 31.3% *p* = 0.74) or the composite endpoint (36.0% vs. 36.8%, *p* = 0.99; Fig. 4A und B). Similarly, TAPSE alone—whether preserved (> 17 mm), intermediate (13–17 mm), or reduced (< 13 mm)—did not predict clinical outcomes (Supplementary Table 4).

## Discussion

This study focused on the prognostic relevance of RV-PA coupling and PAPi in patients with DMR undergoing M-TEER. Main findings of the conducted study are as follows: (I) impaired RV-PA coupling, assessed both echocardiographically and invasively, was associated with increased all-cause mortality and heart failure hospitalization. (II) In contrast, PAPi—despite its prognostic relevance in acute and advanced heart failure—did not predict outcomes in this cohort. (III) These findings suggest that RV-PA coupling, rather than PAPi, provides a more robust marker of RV dysfunction and hemodynamic compromise in patients with chronic left-sided valvular heart disease.

The hypothesis behind this analysis is supported by prior evidence indicating the prognostic impact of RV dysfunction in heart failure [3], pulmonary hypertension [4], and secondary mitral [5] or tricuspid valve disease [7]. RV-PA coupling, typically estimated by the TAPSE/sPAP ratio, reflects the balance between contractile reserve and afterload. This surrogate has demonstrated strong prognostic value in pulmonary hypertension and heart failure with preserved ejection fraction (HFpEF) [10, 11], and more recently in functional MR treated with M-TEER [15, 16]. The current study extends this concept to a DMR population, in which the RV is often considered less affected. Yet, even in the presence of preserved LVEF, significant right heart involvement may exist and carry important prognostic limitations.

In our cohort, mean LV dimensions appeared preserved despite relevant MR. This may reflect patient selection at an earlier disease stage [22], limitations of echocardiographic volumetric quantification in degenerative MR [23, 24], and the multiparametric nature of guideline-based MR grading [2]. Similar findings have been reported in other M-TEER cohorts [18, 25], underscoring the complexity of MR quantification and the necessity of careful integrative echocardiographic assessment.

### Right ventricular dysfunction defined by RV-PA coupling

RV-PA coupling captures the ability of the right ventricle to maintain output in the face of increased afterload. In this study, both echocardiographic (TAPSE/sPAP_echo_ < 0.307 mm/mmHg) and invasive (TAPSE/sPAP_invasive_ < 0.315 mm/mmHg) thresholds were independently associated with worse outcomes. These findings are consistent with prior studies in functional MR [15, 16], pulmonary hypertension [10], and heart failure with preserved ejection fraction [11], which showed that RV-PA uncoupling is associated with increased morbidity and mortality. Importantly, we confirm and extend these findings specifically to degenerative MR, a population with typically preserved LVEF but often underrecognized RV compromise. Right ventricular dysfunction is common in patients with DMR and is associated with increased cardiovascular mortality [6].

Notably, the prognostic cut-offs for RV-PA coupling derived from echocardiography and right heart catheterization were closely aligned—supporting the use of echocardiographic TAPSE/sPAP as a reliable surrogate in most patients. However, the higher sPAP values obtained invasively highlight a potential underestimation of RV afterload when relying solely on Doppler estimates, especially in patients with coexisting TR or suboptimal image quality. The assessment of RV function prior to M-TEER is of clear prognostic importance. In terms of characterization of RV dysfunction single echocardiographic measures have limited diagnostic value, due to the complex contraction pattern of the RV. Although widely used, TAPSE primarily reflects longitudinal shortening of the RV free wall and may not fully capture global RV performance -particularly in the setting of increased afterload.

TAPSE may also be confounded in specific clinical contexts: prior open-heart surgery can reduce longitudinal motion through pericardial adhesions and altered RV geometry [26], while advanced TR may lead to pseudonormalization of TAPSE [27] and underestimation of RV pressures when the valve is no longer restrictive [28]. In our cohort, TR severity was largely comparable between groups, with only a slightly higher proportion of severe TR in the uncoupled group. Importantly, if pseudonormalization had been a dominant factor, these patients would more likely have been classified as coupled. The persistence of worse outcomes in the uncoupled group despite this distribution supports the robustness of RV–PA coupling as an integrative measure of RV function relative to afterload. This observation aligns with previous findings in patients with functional mitral regurgitation (FMR) undergoing M-TEER [29]. Given these limitations, alternative echocardiographic parameters such as fractional area change (FAC) and RV free wall longitudinal strain (RVFWLS) may offer a more comprehensive assessment of RV function [30]. However, these measures are often limited by poor imaging quality and do not incorporate afterload adaptation, which is crucial for understanding RV performance in pressure overload conditions.

Therefore, RV-PA coupling [10–16, 18, 31] and PAPi [20, 32, 33] have been proposed to evaluate RV function as more comprehensive markers of RV function, as they take into account RV contractility relative to afterload.

This simple ratio has been shown to correlate with invasive measures of RV contractile function and has demonstrated prognostic value in various cardiac conditions [10–16, 18, 31]. Several studies in the M-TEER context have used thresholds around 0.35–0.37 mm/mmHg (or the cohort’s median value) to stratify patients [31, 34–36]. However, Doldi et al. identified a threshold of < 0.307 mm/mmHg as the optimal predictor of mortality in a multicenter registry of patients with DMR undergoing M-TEER [18].

In the present study, the prevalence of RV–PA uncoupling was 26.3%, consistent with previously published data in DMR [6, 18, 37] and FMR populations [15, 16]. In this cohort, RV-PA_echo_ uncoupling (TAPSE/sPAP_echo_ < 0.307 mm/mmHg) was associated with higher NYHA functional class, more frequent prior coronary revascularization, and elevated left-sided filling pressures—features indicative of more advanced biventricular remodeling. This aligns with the hemodynamic concept that RV–PA uncoupling may serve as an early integrative marker of circulatory stress—occurring before overt RV systolic failure is evident on TAPSE or RVFAC alone.

Our findings are aligned with previous studies, confirming that RV-PA uncoupling is associated with worse outcomes following M-TEER [15, 18]. Despite similar baseline LVEF, patients with RV-PA uncoupling exhibited higher left atrial pressure (mLAP) and pulmonary capillary wedge pressure (mPCWP), suggesting a greater hemodynamic burden and more advanced disease. In contrast, MR severity was similar between groups, indicating that RV-PA coupling reflects a distinct congestion-related hemodynamic profile [38].

The observation of suboptimal MR reduction in the RV-PA uncoupling group further suggests that advanced disease stage may impair procedural efficacy. These findings reinforce the value of incorporating RV-PA coupling into the preprocedural evaluation to improve risk stratification and patient selection.

### Invasive evaluation of RV function

Despite its widespread use as a surrogate for RV-PA coupling, the echocardiographic TAPSE/sPAP ratio has notable limitations. The assessment of pulmonary pressure via echocardiography, typically derived from tricuspid regurgitation (TR) peak velocity, can be unreliable—particularly in the presence of severe TR [28]. Furthermore, standard estimation of right atrial pressure (RAP) using inferior vena cava diameter may not correlate well with invasively measured RAP [39]. TAPSE itself reflects only longitudinal RV function and may not fully capture global RV performance. In the present study, invasively measured sPAP was, on average, 5 mmHg higher than its echocardiographic estimate, underscoring the complementary role of right heart catheterization (RHC) for accurate hemodynamic assessment. While echocardiographic TAPSE/sPAP ratio is widely used in clinical practice, invasive measurements may offer greater precision, especially in borderline cases or in patients with significant TR.

Previous studies in M-TEER patients have demonstrated greater diagnostic accuracy for invasively measured sPAP and TAPSE/sPAP ratios compared to echocardiographic estimates [40]. However, whether invasive assessment offers clear prognostic superiority in patients with DMR remains uncertain. The gold-standard method for evaluating RV-PA coupling is the ratio of RV end-systolic elastance (Ees) to arterial elastance (Ea), obtained from pressure–volume loop analysis during right heart catheterization [41]. Nonetheless, routine measurements of Ees/Ea remain impractical in the clinical setting due to its technical demands.

In M-TEER studies, RHC is sometimes performed to quantify PH severity and RV filling pressures before intervention [42]. For instance, Koschutnik et al. [31] performed comprehensive invasive hemodynamic assessments alongside echo, though ultimately still defined uncoupling based on the TAPSE/PASP ratio [31]. The cut-off derived for the invasive RV-PA coupling ratio (</≥ 0.315 mm/mmHg), based on Cox regression analysis, closely aligned with the echocardiographic threshold. This concordance supports the validity of echocardiographic assessment alone for RV–PA coupling. The newly identified invasive threshold further validates the role of invasive hemodynamic evaluation in selected patients, especially those with ambiguous echocardiographic data or complex haemodynamics.

To complement our invasive assessment of RV function, we also examined PAPi, defined as (sPAP – dPAP)/RAP. In chronic left-sided heart disease, persistently elevated RAP and pulmonary pressures may reduce PAPi values even when RV function is compensated [43]. While originally developed in the context of acute RV infarction [44] and later validated in LVAD populations [32], PAPi is believed to be more sensitive to acute hemodynamic changes rather than chronic remodeling. In this DMR cohort, PAPi did not predict outcomes. This suggests limited applicability of PAPi in M-TEER patients, whose disease progression is gradual and shaped by chronic compensatory mechanisms. These chronic changes may alter the hemodynamic profile in ways that diminish PAPi’s discriminatory power in this population [44].

Importantly, no association was observed between TAPSE alone and clinical outcomes, further reinforcing that isolated RV function parameters may be insufficient to characterize risk in this population. Prior studies have shown that TAPSE may underestimate RV dysfunction, particularly in chronic pressure overload and in the setting of preserved longitudinal but impaired radial contraction [27, 45]. Instead, RV–PA coupling, by integrating contractility and afterload, provides a more physiologically meaningful measure of right heart performance [10, 46]. This is particularly relevant in DMR, where longstanding MR may lead to subclinical RV dysfunction that is not captured by conventional echocardiographic indices.

These findings carry important clinical implications. RV–PA coupling may serve as a practical tool for preprocedural risk stratification in M-TEER candidates with DMR, identifying patients at elevated risk for postprocedural events. The echocardiographic TAPSE/sPAP ratio is readily obtainable in most patients and may offer prognostic value beyond established surgical risk scores and baseline RV function. In patients with borderline values or conflicting indices, invasive assessment may provide additional clarity. Given its limited prognostic utility in this context, PAPi should be interpreted cautiously and not used as a stand-alone risk marker in patients with DMR undergoing M-TEER.

### Limitations

This study has several limitations. Its retrospective single-center design entails a risk of selection and measurement bias. Follow-up completeness at 5 years was 70%, and although comparable to other long-term registries, loss to follow-up may have introduced bias. Detailed information on mixed MR phenotypes was not systematically available, and patients were classified according to the predominant degenerative mechanism. Invasive haemodynamic measurements were performed under general anesthesia, which may have attenuated absolute values of PAPi and pulmonary pressures; however, efforts were made to maintain hemodynamics close to baseline, and uniform measurement conditions across patients support the internal validity of our findings. Incomplete invasive data and possible variability in echocardiographic sPAP estimates related to TR severity or image quality should also be considered. While the TAPSE/sPAP ratio is widely used, additional coupling measures such as RV strain or RV pressure–volume loop-derived metrics were not available in this dataset. Finally, this study did not evaluate longitudinal changes in RV–PA coupling post-M-TEER, which may provide further insight into recovery patterns and clinical response.

## Conclusion

RV-PA coupling is a strong prognostic marker in DMR patients undergoing M-TEER, reflecting the RV’s adaptive capacity to afterload. Impaired coupling is associated with increased mortality and HFH. In contrast, PAPi lacks prognostic value in this population, likely due to its sensitivity to acute changes and reliance on RAP. Incorporating RV-PA coupling into routine assessment may improve patient selection and guide therapeutic strategies. Future studies should validate these findings in prospective cohorts and explore their integration into clinical practice.

## Supplementary Information

Below is the link to the electronic supplementary material.

ESM1 (DOCX 39.6 KB)

## References

[1] Iung B, Delgado V, Rosenhek R, Price S, Prendergast B, Wendler O et al (2019) Contemporary presentation and management of valvular heart disease: the EURObservational Research Programme Valvular Heart Disease II survey. Circulation 140(14):1156–1169 10.1161/CIRCULATIONAHA.119.041080 31510787

[2] Praz F, Borger MA, Lanz J, Marin-Cuartas M, Abreu A, Adamo M, et al (2025) ESC/EACTS Guidelines for the management of valvular heart disease: Developed by the task force for the management of valvular heart disease of the European Society of Cardiology (ESC) and the European Association for Cardio-Thoracic Surgery (EACTS). Eur Heart J 2025. 10.1093/ejcts/ezaf393 41217787

[3] Meyer P, Filippatos GS, Ahmed MI, Iskandrian AE, Bittner V, Perry GJ et al (2010) Effects of right ventricular ejection fraction on outcomes in chronic systolic heart failure. Circulation 121(2):252–258 10.1161/CIRCULATIONAHA.109.887570 PMC2877272 20048206

[4] Rain S, Handoko ML, Trip P, Gan CT, Westerhof N, Stienen GJ, et al (2013) Right ventricular diastolic impairment in patients with pulmonary arterial hypertension. Circulation 128(18):2016–25, 1–10. 10.1161/CIRCULATIONAHA.113.001873 24056688

[5] Dini FL, Conti U, Fontanive P, Andreini D, Banti S, Braccini L et al (2007) Right ventricular dysfunction is a major predictor of outcome in patients with moderate to severe mitral regurgitation and left ventricular dysfunction. Am Heart J 154(1):172–179 10.1016/j.ahj.2007.03.033 17584573

[6] Le Tourneau T, Deswarte G, Lamblin N, Foucher-Hossein C, Fayad G, Richardson M et al (2013) Right ventricular systolic function in organic mitral regurgitation. Circulation 127(15):1597–1608 10.1161/CIRCULATIONAHA.112.000999 23487435

[7] Dietz MF, Prihadi EA, van der Bijl P, Goedemans L, Mertens BJA, Gursoy E et al (2019) Prognostic implications of right ventricular remodeling and function in patients with significant secondary tricuspid regurgitation. Circulation 140(10):836–845 10.1161/CIRCULATIONAHA.119.039630 31185724

[8] Sanz J, Sánchez-Quintana D, Bossone E, Bogaard HJ, Naeije R (2019) Anatomy, function, and dysfunction of the right ventricle: JACC state-of-the-art review. J Am Coll Cardiol 73(12):1463–1482 10.1016/j.jacc.2018.12.076 30922478

[9] Vonk Noordegraaf A, Westerhof BE, Westerhof N (2017) The relationship between the right ventricle and its load in pulmonary hypertension. J Am Coll Cardiol 69(2):236–243 10.1016/j.jacc.2016.10.047 28081831

[10] Tello K, Wan J, Dalmer A, Vanderpool R, Ghofrani HA, Naeije R et al (2019) Validation of the tricuspid annular plane systolic excursion/systolic pulmonary artery pressure ratio for the assessment of right ventricular-arterial coupling in severe pulmonary hypertension. Circ Cardiovasc Imaging 12(9):e009047 10.1161/CIRCIMAGING.119.009047 PMC7099862 31500448

[11] Guazzi M, Dixon D, Labate V, Beussink-Nelson L, Bandera F, Cuttica MJ, et al (2017) RV Contractile Function and its Coupling to Pulmonary Circulation in Heart Failure With Preserved Ejection Fraction: Stratification of Clinical Phenotypes and Outcomes. JACC Cardiovasc Imaging 10(10 Pt B):1211–21. 10.1016/j.jcmg.2016.12.024 28412423

[12] Brener MI, Lurz P, Hausleiter J, Rodés-Cabau J, Fam N, Kodali SK et al (2022) Right ventricular-pulmonary arterial coupling and afterload reserve in patients undergoing transcatheter tricuspid valve repair. J Am Coll Cardiol 79(5):448–461 10.1016/j.jacc.2021.11.031 35115101

[13] Sugiura A, Tanaka T, Kavsur R, Öztürk C, Silaschi M, Goto T et al (2024) Refining accuracy of RV-PA coupling in patients undergoing transcatheter tricuspid valve treatment. Clin Res Cardiol 113(1):177–186 10.1007/s00392-023-02339-5 PMC10808486 38010521

[14] Sultan I, Cardounel A, Abdelkarim I, Kilic A, Althouse AD, Sharbaugh MS et al (2019) Right ventricle to pulmonary artery coupling in patients undergoing transcatheter aortic valve implantation. Heart 105(2):117–121 10.1136/heartjnl-2018-313385 PMC6320315 30093545

[15] Karam N, Stolz L, Orban M, Deseive S, Praz F, Kalbacher D et al (2021) Impact of right ventricular dysfunction on outcomes after transcatheter edge-to-edge repair for secondary mitral regurgitation. JACC Cardiovasc Imaging 14(4):768–778 10.1016/j.jcmg.2020.12.015 33582067

[16] Brener MI, Grayburn P, Lindenfeld J, Burkhoff D, Liu M, Zhou Z et al (2021) Right ventricular-pulmonary arterial coupling in patients with HF secondary MR: analysis from the COAPT trial. JACC Cardiovasc Interv 14(20):2231–2242 10.1016/j.jcin.2021.07.047 34674862

[17] Zoghbi WA, Adams D, Bonow RO, Enriquez-Sarano M, Foster E, Grayburn PA et al (2017) Recommendations for noninvasive evaluation of native valvular regurgitation: a report from the American Society of Echocardiography developed in collaboration with the Society for Cardiovascular Magnetic Resonance. J Am Soc Echocardiogr 30(4):303–371 10.1016/j.echo.2017.01.007 28314623

[18] Doldi PM, Stolz L, Kalbacher D, Köll B, Geyer M, Ludwig S et al (2022) Right ventricular dysfunction predicts outcome after transcatheter mitral valve repair for primary mitral valve regurgitation. Eur J Heart Fail 24(11):2162–2171 10.1002/ejhf.2661 36054557

[19] Sciomer S, Magrì D, Badagliacca R (2007) Non-invasive assessment of pulmonary hypertension: Doppler-echocardiography. Pulm Pharmacol Ther 20(2):135–140 10.1016/j.pupt.2006.03.008 16753319

[20] Kochav SM, Flores RJ, Truby LK, Topkara VK (2018) Prognostic impact of pulmonary artery pulsatility index (PAPi) in patients with advanced heart failure: insights from the ESCAPE trial. J Card Fail 24(7):453–459 10.1016/j.cardfail.2018.03.008 29597051

[21] Contal C, O’Quigley J (1999) An application of changepoint methods in studying the effect of age on survival in breast cancer. Comput Stat Data Anal 30(3):253–70. 10.1016/S01679473(98)000966

[22] Gaasch WH, Meyer TE (2008) Left ventricular response to mitral regurgitation. Circulation 118(22):2298–2303 10.1161/CIRCULATIONAHA.107.755942 19029478

[23] Uretsky S, Gillam L, Lang R, Chaudhry FA, Argulian E, Supariwala A et al (2015) Discordance Between Echocardiography and MRI in the Assessment of Mitral Regurgitation Severity: A Prospective Multicenter Trial. J Am Coll Cardiol 65(11):1078–1088 10.1016/j.jacc.2014.12.047 25790878

[24] Lee K, Om SY, Lee SH, Oh JK, Park H-K, Choi Y-S et al (2020) Clinical situations associated with inappropriately large regurgitant volumes in the assessment of mitral regurgitation severity using the proximal flow convergence method in patients with chordae rupture. J Am Soc Echocardiogr 33(1):64–71 10.1016/j.echo.2019.08.020 31668504

[25] Ludwig S, Koell B, Weimann J, Donal E, Patel D, Stolz L et al (2024) Impact of intraprocedural mitral regurgitation and gradient following transcatheter edge-to-edge repair for primary mitral regurgitation. JACC Cardiovasc Interv 17(13):1559–1573 10.1016/j.jcin.2024.05.018 38986655

[26] Tamborini G, Muratori M, Brusoni D, Celeste F, Maffessanti F, Caiani EG et al (2009) Is right ventricular systolic function reduced after cardiac surgery? A two- and three-dimensional echocardiographic study. Eur J Echocardiogr 10(5):630–634 10.1093/ejechocard/jep015 19252190

[27] Prihadi EA, van der Bijl P, Dietz M, Abou R, Vollema EM, Marsan NA et al (2019) Prognostic implications of right ventricular free wall longitudinal strain in patients with significant functional tricuspid regurgitation. Circ Cardiovasc Imaging 12(3):e008666 10.1161/CIRCIMAGING.118.008666 30879327

[28] Özpelit E, Akdeniz B, Özpelit EM, Tas S, Alpaslan E, Bozkurt S et al (2015) Impact of severe tricuspid regurgitation on accuracy of echocardiographic pulmonary artery systolic pressure estimation. Echocardiography 32(10):1483–1490 10.1111/echo.12912 25735427

[29] Lupi L, Italia L, Pagnesi M, Pancaldi E, Ancona F, Stella S et al (2023) Prognostic value of right ventricular longitudinal strain in patients with secondary mitral regurgitation undergoing transcatheter edge-to-edge mitral valve repair. European Heart Journal - Cardiovascular Imaging 24(11):1509–1517 10.1093/ehjci/jead103 37194460

[30] Hahn RT, Lerakis S, Delgado V, Addetia K, Burkhoff D, Muraru D et al (2023) Multimodality Imaging of Right Heart Function: JACC Scientific Statement. J Am Coll Cardiol 81(19):1954–1973 10.1016/j.jacc.2023.03.392 37164529

[31] Koschutnik M, Donà C, Nitsche C, Kammerlander AA, Dannenberg V, Brunner C et al (2025) Impact of right ventricular-to-pulmonary artery coupling on remodeling and outcome in patients undergoing transcatheter edge-to-edge mitral valve repair. Clin Res Cardiol 114(2):156–167 10.1007/s00392-023-02318-w PMC11839846 37870628

[32] Kang G, Ha R, Banerjee D (2016) Pulmonary artery pulsatility index predicts right ventricular failure after left ventricular assist device implantation. J Heart Lung Transplant 35(1):67–73 10.1016/j.healun.2015.06.009 26212656

[33] Lim Y, Low TT, Chan SP, Lin W, Teo TW, Jang JJ et al (2021) Does pulmonary artery pulsatility index predict mortality in pulmonary arterial hypertension? ESC Heart Fail 8(5):3835–3844 10.1002/ehf2.13450 PMC8497387 34165259

[34] Shechter A, Vaturi M, Kaewkes D, Koren O, Koseki K, Solanki A et al (2023) Prognostic value of baseline tricuspid annular plane systolic excursion to pulmonary artery systolic pressure ratio in mitral transcatheter edge-to-edge repair. J Am Soc Echocardiogr 36(4):391-401.e19 10.1016/j.echo.2022.12.026 36657500

[35] Trejo-Velasco B, Estevez-Loureiro R, Carrasco-Chinchilla F, Fernández-Vázquez F, Arzamendi D, Pan M et al (2021) Prognostic role of TAPSE to PASP ratio in patients undergoing MitraClip procedure. J Clin Med. 10.3390/jcm10051006 PMC7958333 33801311

[36] Popolo Rubbio A, Testa L, Granata G, Salvatore T, De Marco F, Casenghi M et al (2022) Prognostic significance of right ventricle to pulmonary artery coupling in patients with mitral regurgitation treated with the MitraClip system. Catheter Cardiovasc Interv 99(4):1277–1286 10.1002/ccd.30044 34939726

[37] Bohbot Y, Essayagh B, Benfari G, Bax JJ, Le Tourneau T, Topilsky Y et al (2025) Prognostic implications of right ventricular dysfunction in severe degenerative mitral regurgitation. J Am Heart Assoc 14(1):e036206 10.1161/JAHA.124.036206 PMC12054403 39692024

[38] Bakkestrøm R, Banke A, Christensen NL, Pecini R, Irmukhamedov A, Andersen M et al (2018) Hemodynamic characteristics in significant symptomatic and asymptomatic primary mitral valve regurgitation at rest and during exercise. Circ Cardiovasc Imaging 11(2):e007171 10.1161/CIRCIMAGING.117.007171 29449412

[39] Brennan JM, Blair JE, Goonewardena S, Ronan A, Shah D, Vasaiwala S et al (2007) Reappraisal of the use of inferior vena cava for estimating right atrial pressure. J Am Soc Echocardiogr 20(7):857–861 10.1016/j.echo.2007.01.005 17617312

[40] Masiero G, Arturi F, Soramio EB, Fovino LN, Fabris T, Cardaioli F et al (2025) Impact of updated invasive right ventricular and pulmonary hemodynamics on long-term outcomes in patients with mitral valve transcatheter edge-to-edge repair. Am J Cardiol 234:99–106 10.1016/j.amjcard.2024.11.010 39572265

[41] Vonk-Noordegraaf A, Haddad F, Chin KM, Forfia PR, Kawut SM, Lumens J et al (2013) Right heart adaptation to pulmonary arterial hypertension. J Am Coll Cardiol 62(25_Supplement):D22–D33 10.1016/j.jacc.2013.10.027 24355638

[42] Jaeger P, Toskas I, Henes J-K, Shcherbyna S, Schwarz F, Euper M et al (2025) Impact of pulmonary hypertension on outcomes after TEER in patients suffering from mitral regurgitation. Clin Res Cardiol 114(2):203–214 10.1007/s00392-024-02442-1 PMC11839688 38565712

[43] Lim HS, Gustafsson F (2020) Pulmonary artery pulsatility index: physiological basis and clinical application. Eur J Heart Fail 22(1):32–38 10.1002/ejhf.1679 31782244

[44] Korabathina R, Heffernan KS, Paruchuri V, Patel AR, Mudd JO, Prutkin JM et al (2012) The pulmonary artery pulsatility index identifies severe right ventricular dysfunction in acute inferior myocardial infarction. Catheter Cardiovasc Interv 80(4):593–600 10.1002/ccd.23309 21954053

[45] Ogawa M, Kuwajima K, Yamane T, Hasegawa H, Yagi N, Shiota T (2024) Prognostic implication of right ventricular free wall longitudinal strain and right atrial pressure estimated by echocardiography in patients with severe functional tricuspid regurgitation. J Am Heart Assoc 13(8):e033196 10.1161/JAHA.123.033196 PMC11262525 38609840

[46] Guazzi M, Bandera F, Pelissero G, Castelvecchio S, Menicanti L, Ghio S et al (2013) Tricuspid annular plane systolic excursion and pulmonary arterial systolic pressure relationship in heart failure: an index of right ventricular contractile function and prognosis. American Journal of Physiology-Heart and Circulatory Physiology 305(9):H1373–H1381 10.1152/ajpheart.00157.2013 23997100

