# Supplementary material for: Echocardiographic and invasive RV-PA coupling predict mortality after edge-to-edge repair for degenerative mitral regurgitation, while pulmonary artery pulsatility index does not: a single-center observational study
Source: Clin Res Cardiol. 2025 Dec 9;115(12):2161–71. doi: 10.1007/s00392-025-02795-1 (PMC13751578; doi:10.1007/s00392-025-02795-1)
Supplement: Supplementary file 1 [file 392_2025_2795_MOESM1_ESM.docx]

**Supplementary Tables**

| Supplementary Table 1. Study baseline characteristics according to whole patient cohort | |
| --- | --- |
|  | **All patients (n=293)** |
| Clinical data |  |
| Male sex, n (%) | 153 (52.2) |
| Age, years | 81.0 [76.9-84.2] |
| BMI, kg/cm^2^ | 24.7 [22.5-28.0] |
| STS Score, % | 3.9 [2.7-5.4] |
| NYHA class |  |
| I | 2 (0.8) |
| II | 29 (11.7) |
| III | 163 (65.7) |
| IV | 54 (21.8) |
| Cardiovascular risk factors |  |
| AHT, n (%) | 219 (84.2) |
| Dyslipidemia, n (%) | 95 (42.0) |
| Nicotine abuse, n (%) | 54 (28.3) |
| DM, n (%) | 45 (16.8) |
| Comorbidities |  |
| CAD, n (%) | 172 (65.9) |
| ICM, n (%) | 58 (40.0) |
| Previous MI, n (%) | 31 (14.4) |
| Previous PCI, n (%) | 103 (43.5) |
| Previous CABG, n (%) | 37 (16.7) |
| Previous ICD/CRT | 33 (15.6) |
| COPD, n (%) | 53 (19.4) |
| Previous Stroke, n (%) | 28 (12.8) |
| Afib | 216 (79.1) |
| GFR (ml/min for 1.73 m^2^) | 46.2 [32.8-62.2] |
| NT-proBNP (pg/ml) | 2837  [1407-5354] |
| Echocardiographic data |  |
| LVEF, % | 54.5 [46.3-60.4] |
| LVESV (Simpsons), ml | 48.8 [35.0-70.0] |
| LVEDV (Simpsons), ml | 106.3  [81.1-142.8] |
| TAPSE, mm | 18.0 [15.0-22.0] |
| sPAP_echo_, mmHg | 43.0 [34.0-55.0] |
| MR grade |  |
| 2+ | - |
| 3+ | 46 (17.3) |
| 4+ | 217 (81.53) |
| TR grade |  |
| 0+ | 3 (1.4) |
| 1+ | 75 (35.6) |
| 2+ | 62 (29.4) |
| 3+ | 41 (19.4) |
| 4+ | 30 (14.2) |
| Haemodynamic data (RHC) |  |
| sPAP_RHC_, mmHg | 47.0 [37.0-55.0] |
| dPAP_RHC_, mmHg | 18.0 [13.0-22.0] |
| mPAP_RHC_, mmHg | 29.0 [22.9-35.0] |
| mPCWP_RHC_, mmHg | 18.0 [13.0-23.0] |
| mLAP_RHC_, mmHg | 15.0 [12.0-21.0] |
| CVP_RHC_, mmHg | 8.0 [6.0-11.0] |

AHT = arterial hypertension; Afib = atrial fibrillation, BMI = body mass index; CABG = coronary artery bypass graft, CAD = coronary artery disease, COPD = chronic obstructive pulmonary disease, CRT = cardiac resynchronization therapy, CVP = central venous pressure, DM = diabetes mellitus, dPAP = diastolic pulmonary artery pressure, echo = echocardiography, GFR = glomerular filtration rate, ICD = implantable cardioverter-defibrillator, ICM = ischemic heart disease, LVEDV = left ventricular diastolic volume, LVEF = left ventricular ejection fraction, LVESV = left ventricular systolic volume, MI = myocardial infarction, mLAP = mean left atrial pressure, mPAP = mean pulmonary artery pressure, mPCWP = pulmonary capillary wedge pressure, MR = mitral regurgitation, NT-proBNP = N-terminal pro-b-type natriuretic peptide, NYHA = New York Heart Association, PCI = percutaneous coronary intervention, RHC = right heart catheter, sPAP = systolic pulmonary artery pressure, STS Score = Society of Throacic Surgeons Score, TAPSE = tricuspid annular plane systolic excursion, TR = tricuspid regurgitation

| Supplementary Table 2. Study baseline characteristics according to RV-PA Coupling Invasive | | | | | | |
| --- | --- | --- | --- | --- | --- | --- |
|  | **All patients (n=100)** | **RV-PA Invasive ≥0.315**  **(n=70)** | | **RV-PA Invasive <0.315**  **(n=30)** | | **p-value** |
| Clinical data |  | |  |  |  | |
| Male sex, n (%) | 53 (53.0) | | 35 (50.0) | 18 (60.0) | 0.21 | |
| Age, years | 80.5 [76.5-84.6] | | 81.1 [77.7-85.3] | 79.8 [73.8-83.9] | 0.48 | |
| BMI, kg/cm^2^ | 24.8 [22.2-28.1] | | 24.8 [22.8-28.1] | 24.8 [21.7-28.5] | 0.61 | |
| STS Score, % | 4.1 [2.8-5.8] | | 4.0 [3.0-5.7] | 4.9 [2.5-6.7] | 0.87 | |
| NYHA class |  | |  |  |  | |
| I | 1 (1.2) | | 1 (1.7) | - | 1.00 | |
| II | 13 (14.9) | | 10 (17.0) | 3 (10.7) | 0.66 | |
| III | 53 (60.9) | | 36 (61.0) | 17 (60.7) | 1.00 | |
| IV | 20 (23.0) | | 12 (20.3) | 8 (28.6) | 0.56 | |
| Cardiovascular risk factors |  | |  |  |  | |
| AHT, n (%) | 178 (84.8) | | 55 (85.9) | 23 (82.1) | 0.88 | |
| Dyslipidemia, n (%) | 31 (40.3) | | 20 (39.2) | 11 (42.3) | 0.99 | |
| Nicotine abuse, n (%) | 18 (28.0) | | 20 (27.0) | 9 (30.0) | 0.95 | |
| DM, n (%) | 32 (31.6) | | 12 (30.0) | 6 (35.3) | 0.93 | |
| Comorbidities |  | |  |  |  | |
| CAD, n (%) | 60 (66.7) | | 39 (63.9) | 21 (72.4) | 0.58 | |
| ICM, n (%) | 21 (30.8) | | 14 (31.1) | 7 (30.4) | 1.00 | |
| Previous MI, n (%) | 6 (8.1) | | 2 (4.1) | 4 (16.0) | 0.18 | |
| Previous PCI, n (%) | 39 (47.0) | | 25 (44.6) | 14 (51.9) | 0.70 | |
| Previous CABG, n (%) | 12 (16.0) | | 6 (11.8) | 6 (25.0) | 0.26 | |
| Previous ICD/CRT | 17 (23.0) | | 11 (22.0) | 6 (25.0) | 1.00 | |
| COPD, n (%) | 21 (21.9) | | 13 (29.7) | 8 (26.7) | 0.62 | |
| Previous Stroke, n (%) | 9 (12.0) | | 7 (14.0) | 2 (8.0) | 0.71 | |
| Afib | 76 (79.1) | | 52 (78.8) | 24 (80.0) | 1.00 | |
| GFR (ml/min for 1.73 m^2^) | 46.3 [33.5-63.2] | | 48.2 [37.7-63.6] | 42.9 [32.1-63.2] | 0.43 | |
| NT-proBNP (pg/ml) | 3379  [1840-5137] | | 3236  [1433-4918] | 4257  [2612-8152] | 0.074 | |
| Echocardiographic data |  | |  |  |  | |
| LVEF, % | 53.3 [44.2-58.2] | | 54.1 [49.0-59.2] | 50.0 [37.9-56.1] | 0.058 | |
| LVESV (Simpsons), ml | 50.0 [37.8-70.0] | | 50.3 [38.7-69.6] | 49.1 [35.3-86.8] | 0.76 | |
| LVEDV (Simpsons), ml | 106.5  [80.9-139.7] | | 106.3  [90.0-138.3] | 107.0  [77.3-147.3] | 0.62 | |
| TAPSE, mm | 17.9 [14.0-20.0] | | 19.0 [17.0-22.1] | 14.0 [11.0-15.1] | <0.001 | |
| sPAP_echo_, mmHg | 45.0 [36.0-55.2] | | 44.0 [34.7-55.3] | 49.0 [40.0-55.3] | 0.20 | |
| MR grade |  | |  |  |  | |
| 2+ | - | | - | - | - | |
| 3+ | 24 (24.7) | | 18 (26.5) | 6 (20.7) | 0.73 | |
| 4+ | 73 (75.3) | | 50 (73.5) | 23 (79.3) | 0.73 | |
| TR grade |  | |  |  |  | |
| 0+ | 1 (1.1) | | 1 (1.6) | - | - | |
| 1+ | 30 (33.7) | | 23 (37.7) | 7 (25.0) | 0.35 | |
| 2+ | 27 (30.3) | | 19 (31.1) | 8 (28.6) | 1.00 | |
| 3+ | 18 (20.2) | | 9 (14.8) | 9 (32.1) | 0.11 | |
| 4+ | 13(14.6) | | 9 (14.8) | 4 (14.3) | 1.00 | |
| Haemodynamic data (RHC) |  | |  |  |  | |
| sPAP_RHC_, mmHg | 47.0 [38.4-54.0] | | 43.5 [34.9-49.0] | 54.0 [47.9-65.3] | <0.001 | |
| dPAP_RHC_, mmHg | 19.0 [14.0-21.6] | | 16.0 [14.0-20.0] | 22.0 [19.0-24.0] | <0.001 | |
| mPAP_RHC_, mmHg | 29.0 [24.0-33.6] | | 27.5 [22.0-30.0] | 34.5 [24.0-33.6] | <0.001 | |
| mPCWP_RHC_, mmHg | 18.0 [13.2-23.0] | | 15.0 [11.0-22.0] | 23.0 [19.8-26.1] | <0.001 | |
| mLAP_RHC_, mmHg | 15.5 [12.0-21.6] | | 14.0 [11.0-19.0] | 20.5 [15.0-25.1] | <0.001 | |
| CVP_RHC_, mmHg | 8.0 [6.0-11.0] | | 8.0 [5.0-10.0] | 9.5 [7.9-13.1] | 0.01 | |
| AHT = arterial hypertension; Afib = atrial fibrillation, BMI = body mass index; CABG = coronary artery bypass graft, CAD = coronary artery disease, COPD = chronic obstructive pulmonary disease, CRT = cardiac resynchronization therapy, CVP = central venous pressure, DM = diabetes mellitus, dPAP = diastolic pulmonary artery pressure, echo = echocardiography, GFR = glomerular filtration rate, ICD = implantable cardioverter-defibrillator, ICM = ischemic heart disease, LVEDV = left ventricular diastolic volume, LVEF = left ventricular ejection fraction, LVESV = left ventricular systolic volume, MI = myocardial infarction, mLAP = mean left atrial pressure, mPAP = mean pulmonary artery pressure, mPCWP = pulmonary capillary wedge pressure, MR = mitral regurgitation, NT-proBNP = N-terminal pro-b-type natriuretic peptide, NYHA = New York Heart Association, PCI = percutaneous coronary intervention, RHC = right heart catheter, sPAP = systolic pulmonary artery pressure, STS Score = Society of Throacic Surgeons Score, TAPSE = tricuspid annular plane systolic excursion, TR = tricuspid regurgitation | | | | | | |

| Supplementary Table 3. Study baseline characteristics according to PAPi | | | | | | |
| --- | --- | --- | --- | --- | --- | --- |
|  | **All patients (n=210)** | **PAPi ≥4.2**  **(n=77)** | | **PAPi <4.2**  **(n=133)** | | **p-value** |
| Clinical data |  | |  |  |  | |
| Male sex, n (%) | 114 (54.3) | | 38 (49.4) | 76 (57.1) | 0.34 | |
| Age, years | 81.0 [76.5-84.0] | | 80.1 [76.0-83.5] | 81.4 [77.3-84.1] | 0.25 | |
| BMI, kg/cm^2^ | 24.7 [22.2-28.5] | | 24.4 [21.3-26.5] | 25.7 [22.8-29.5] | 0.013 | |
| STS Score, % | 3.9 [2.7-5.4] | | 4.0 [2.5-5.1] | 3.9 [2.8-5.7] | 0.77 | |
| NYHA class |  | |  |  |  | |
| I | 1 (0.6) | | - | 1 (0.9) | 1.00 | |
| II | 23 (13.5) | | 10 (15.9) | 13 (12.0) | 0.63 | |
| III | 107 (62.6) | | 39 (61.9) | 68 (63.0) | 1.00 | |
| IV | 40 (23.4) | | 14 (22.2) | 26 (24.1) | 0.93 | |
| Cardiovascular risk factors |  | |  |  |  | |
| AHT, n (%) | 157 (82.6) | | 56 (81.2) | 101 (83.5) | 0.84 | |
| Dyslipidemia, n (%) | 63 (38.2) | | 24 (39.3) | 39 (37.5) | 0.94 | |
| Nicotine abuse, n (%) | 40 (29.4) | | 13 (28.3) | 27 (30.0) | 0.99 | |
| DM, n (%) | 30 (15.3) | | 12 (16.2) | 18 (14.8) | 0.94 | |
| Comorbidities |  | |  |  |  | |
| CAD, n (%) | 120 (64.9) | | 48 (71.6) | 72 (61.0) | 0.20 | |
| ICM, n (%) | 36 (35.6) | | 9 (27.3) | 27 (39.7) | 0.32 | |
| Previous MI, n (%) | 21 (13.4) | | 8 (13.6) | 13 (13.3) | 1.00 | |
| Previous PCI, n (%) | 73 (42.4) | | 25 (38.5) | 48 (44.9) | 0.51 | |
| Previous CABG, n (%) | 23 (14.5) | | 8 (13.6) | 15 (15.0) | 0.99 | |
| Previous ICD/CRT | 30 (19.4) | | 11 (19.0) | 19 (19.6) | 1.00 | |
| COPD, n (%) | 38 (19.4) | | 12 (16.2) | 26 (21.3) | 0.49 | |
| Previous Stroke, n (%) | 18 (11.4) | | 5 (8.3) | 13 (13.3) | 0.49 | |
| Afib | 158 (80.6) | | 58 (78.4) | 100 (82.0) | 0.67 | |
| GFR (ml/min for 1.73 m^2^) | 44.4 [32.3-61.9] | | 44.4 [33.7-64.1] | 44.5 [31.5-59.9] | 0.69 | |
| NT-proBNP (pg/ml) | 3127  [1457-6051] | | 2434  [799-6060] | 3346  [1915-6122] | 0.11 | |
| Echocardiographic data |  | |  |  |  | |
| LVEF, % | 53.5 [44.6-59.0] | | 54.5 [47.6-58.9] | 53.2 [44.3-59.6] | 0.72 | |
| LVESV (Simpsons), ml | 49.1 [37.2-70.0] | | 50.0 [38.2-70.0] | 49.1 [35.2-70.5] | 0.91 | |
| LVEDV (Simpsons), ml | 107.0  [80.8-143.5] | | 106.1  [83.0-153.1] | 107.3  [80.0-140.2] | 0.71 | |
| TAPSE, mm | 17.7 [14.0-20.0] | | 19.0 [16.8-21.6] | 17.0 [14.0-20.0] | <0.023 | |
| sPAP_echo_, mmHg | 45.0 [35.4-55.0] | | 50.0 [35.0-63.6] | 42.0 [36.0-53.6] | 0.14 | |
| MR grade |  | |  |  |  | |
| 2+ | - | | - | - | - | |
| 3+ | 35 (19.0) | | 13 (19.7) | 22 (18.6) | 1.00 | |
| 4+ | 146 (79.4) | | 52 (78.8) | 94 (79.7) | 1.00 | |
| TR grade |  | |  |  |  | |
| 0+ | 3 (2.3) | | 3 (6.5) | - | - | |
| 1+ | 46 (35.1) | | 14 (30.4) | 32 (37.7) | 0.53 | |
| 2+ | 34 (26.0) | | 16 (34.8) | 18 (21.2) | 0.14 | |
| 3+ | 27 (20.6) | | 7 (15.2) | 20 (23.5) | 0.37 | |
| 4+ | 21(16.0) | | 6 (13.0) | 15 (17.7) | 0.66 | |
| Haemodynamic data (RHC) |  | |  |  |  | |
| sPAP_RHC_, mmHg | 47.5 [37.0-55.0] | | 49.0 [39.3-62.3] | 44.0 [36.7-53.0] | 0.0062 | |
| dPAP_RHC_, mmHg | 18.0 [13.0-22.0] | | 16.0 [12.0-20.3] | 19.0 [14.0-23.0] | <0.001 | |
| mPAP_RHC_, mmHg | 29.0 [22.9-35.0] | | 30.0 [22.0-34.3] | 28.0 [23.0-35.0] | 0.98 | |
| mPCWP_RHC_, mmHg | 18.0 [13.0-23.0] | | 16.0 [10.1-23.0] | 18.0 [14.2-23.0] | 0.045 | |
| mLAP_RHC_, mmHg | 15.0 [12.0-20.3] | | 14.0 [10.0-19.0] | 16.0 [12.0-21.6] | 0.012 | |
| CVP_RHC_, mmHg | 8.0 [6.0-11.0] | | 6.0 [4.0-7.3] | 10.0 [8.0-13.0] | <0.001 | |
| AHT = arterial hypertension; Afib = atrial fibrillation, BMI = body mass index; CABG = coronary artery bypass graft, CAD = coronary artery disease, COPD = chronic obstructive pulmonary disease, CRT = cardiac resynchronization therapy, CVP = central venous pressure, DM = diabetes mellitus, dPAP = diastolic pulmonary artery pressure, echo = echocardiography, GFR = glomerular filtration rate, ICM = ischemic heart disease, LVEDV = left ventricular diastolic volume, LVEF = left ventricular ejection fraction, LVESV = left ventricular systolic volume, , ICD = implantable cardioverter-defibrillator, , MI = myocardial infarction, mLAP = mean left atrial pressure, mPAP = mean pulmonary artery pressure, mPCWP = pulmonary capillary wedge pressure, MR = mitral regurgitation, NT-proBNP = N-terminal pro-b-type natriuretic peptide, NYHA = New York Heart Association, PAPi = pulmonary artery pulsatility index, PCI = percutaneous coronary intervention, RHC = right heart catheter, sPAP = systolic pulmonary artery pressure, STS Score = Society of Throacic Surgeons Score, TAPSE = tricuspid annular plane systolic excursion, TR = tricuspid regurgitation | | | | | | |

**Supplementary Table 4: Impact of RV-function assessed by categorized TAPSE on patient outcomes**

| 2 years all-cause mortality | HR (95% CI) | HR per SD (95% CI) | p-value |
| --- | --- | --- | --- |
| TAPSE <13 mm  TAPSE 13-17 mm  TAPSE >17 mm | reference  0.85 (0.38, 1.89)  0.49 (0.23, 1.06) | 0.93 (0.65, 1.33)  0.70 (0.48, 1.03) | 0.101  0.69  0.072 |
|  |  |  |  |
| 2 years all-cause mortality or HFH | **HR (95% CI)** | **HR per SD (95% CI)** | **p-value** |
| TAPSE <13 mm  TAPSE 13-17 mm  TAPSE >17 mm | reference  0.93 (0.43, 1.98)  0.66 (0.32, 1.34) | 0.97 (0.69, 1.36)  0.81 (0.57, 1.16) | 0.357  0.85  0.25 |
| CI = confidence interval, HFH = heart failure hospitalization, HR = hazard ratio, SD = standard deviation, TAPSE = tricuspid annular plane systolic excursion | | | |
